# Staining Analysis of Resin Cements and Their Effects on Colour and Translucency Changes in Lithium Disilicate Veneers

**DOI:** 10.3390/polym17030362

**Published:** 2025-01-29

**Authors:** Vesna Miletic, Asana Pour Ronagh

**Affiliations:** Faculty of Medicine and Health, Sydney Dental School, The University of Sydney, Surry Hills, NSW 2010, Australia; asana.pourronagh@sydney.edu.au

**Keywords:** resin cement, lithium disilicate glass ceramic, veneer, colour, staining, CIEDE2000

## Abstract

This study evaluated the colour and translucency changes in resin cements and lithium disilicate veneer restorations, as well as the sorption and solubility of cements after staining. Four resin cements (G-CEM ONE, RelyX Universal, Panavia V5, Variolink Esthetic) were tested. Cylindrical specimens and LiSi veneer restorations cemented to a G-aenial Universal Injectable dentine base (N = 10/group) were stained in black tea for 72 h. Changes in colour (*∆E_00_*) and the translucency parameter (*∆TP_00_*) were analyzed using a spectrophotometer, while sorption and solubility were calculated via mass/volume formulae. G-Cem and RelyX exhibited significantly lower *∆E_00_* than Variolink and Panavia (*p* < 0.001), while RelyX uniquely showed increased *∆TP_00_* (*p* < 0.001). The *∆E_00_* of cemented veneers ranged from 2.7 ± 0.5 (G-Cem) to 3.9 ± 0.7 (Panavia), with decreased translucency after staining across groups (*p* > 0.05). The initial TP positively correlated with the *∆E_00_* of both cements and veneer restorations, while the *∆E_00_* of cements correlated with that of veneer restorations. RelyX had the highest sorption, and Variolink showed the highest solubility, though neither correlated with colour changes. Resin cements influenced colour changes in veneer restorations post-staining, with colour differences associated with initial cement translucency but independent of sorption and solubility.

## 1. Introduction

Ceramic veneers are widely accepted as a minimally invasive restorative option for anterior teeth, along with direct composite veneers. Although direct composite veneers require less tissue preparation, they are more susceptible to discolouration and the loss of surface gloss [1]. Feldspathic, leucite-reinforced glass ceramic and lithium disilicate ceramic show favourable long-term results with lithium disilicate ceramic being somewhat superior from the technical and biological perspective [2]. Aesthetic complications related to veneers increase over time, and marginal discolouration appears a frequent problem regardless of the type of ceramic [2].

Resin cements have good mechanical and aesthetic properties, good handling characteristics and a long-lasting bond between ceramic restorations and the remaining tooth tissue [3]. Two important characteristics of resin cements have led the development of these materials, one focusing on adhesion and the other on polymerization. Self-adhesive resin cements simplify the procedure; however, conflicting evidence exists regarding their bonding efficiency to dentine when compared to multi-step adhesive systems [4,5]. Dual-cured resin cements utilize photopolymerization, allowing fast and predictable restoration stabilization, whilst, at the same time, improving the degree of conversion in inaccessible regions due to chemical polymerization [3].

Optical properties of resin cements, regardless of their type, are subject to changes, which may affect the optical properties of ceramic restorations, either initially [6,7,8] or after ageing [9,10,11,12,13,14] or staining [15,16,17,18]. Dual-cured resin cements showed greater colour changes after thermal cyclic ageing compared to light-curing resin-based materials used for luting ceramic veneers [9,10]. The discolouration of dual-cured resin cements was attributed to the oxidation of amine co-initiators [14]. Cement types were associated with colour differences in various ceramic CAD/CAM materials [7,8] as were cement thickness [8], shade [10] and substrate shade [7]. Changes in optical properties of cemented veneers were related to the type of cement [10,14,17], inorganic filler content [6] and the presence of amine co-initiators [14] but not to the type of light-curing unit [12]. 

Large variations in methodological approaches exist among previous studies. Whilst, in many studies, specimens were prepared of resin cements alone [11,15,16,17,18,19,20,21], in others, optical changes were determined when ceramic restorations were cemented to a resin composite base [6,7,8,10] or bovine teeth [9,12].

Feldspathic porcelain veneers were used in previous studies focusing on the effects of resin cements on the optical properties of veneers, [6,8,9,10]. Lithium disilicate ceramic veneers were used with a single resin cement to test the effect of light-curing protocols [12] or with light-cured resin cements to determine their effects on initial colour and translucency [7]. There is insufficient evidence on the effects of resin cements on optical properties of lithium disilicate veneers, particularly after staining. Moreover, it is unclear if any relationship exists between optical properties and other factors, such as sorption and solubility of cements.

The aims of this study were to determine colour and translucency changes, the sorption and solubility of resin cements following staining and their effects on cemented lithium disilicate veneers and to investigate possible relationships between cement properties. The null hypotheses were as follows: (1) there are no statistically significant differences in colour and translucency changes, the sorption and solubility of resin cements after staining; (2) there are no statistically significant differences in colour and translucency changes in cemented lithium disilicate veneers after staining; (3) there is no significant relationship between the colour or translucency of resin cements and cemented lithium disilicate veneers and (4) there is no significant relationship between the sorption and solubility of resin cements and the colour or translucency of cemented lithium disilicate veneers.

## 2. Materials and Methods

G* Power analysis was conducted with the following input parameters: F Test family, ANOVA fixed effects, main effects and interactions, effect size (large) = 0.4, alpha = 0.5, power = 0.8, numerator *df* = 3, number of groups = 8. This analysis revealed that the sample size per group was 9.125, resulting in the decision to use 10 specimens per group.

Table 1 provides details of the materials used in this study [22]. Two types of specimens were prepared: (1) resin cements only—to test colour and translucency changes, sorption and solubility following staining and (2) cemented lithium disilicate veneers—to test the colour and translucency changes in veneers cemented with different resin cements following staining.

### 2.1. Specimen Preparation

Specimens of GC Cem, RelyX, Panavia and Variolink were prepared in standardized 3D-printed cylindrical moulds (10 × 2 mm). A mould was placed on top of a glass slab, and cement was injected by submerging the tip into the material, extruding the material at a slow pace and carefully covering with a celluloid strip to avoid the entrapment of air voids. The material was light-cured for 10 s using a high-intensity unit (SmartLite Pro, Dentsply Sirona, Charlotte, NC, USA), at a distance of 1 mm, which was maintained with a peripheral spacer, also maintaining horizontal positioning of the light tip (∅10 mm), parallel to the specimen surface. Light irradiance and spectral distribution of the light-curing unit were monitored using a spectrophotometer (MARC, BlueLight Analytics, Halifax, NS, Canada). Specimens were prepared by one operator (APR) and wet-polished using a series of sof-lex discs, each for 30 s.

Another set of LiSi veneers were cemented to the G-aenial composite used as a standardized dentine base (N = 10/group). The Initial LiSi Block was cut into specimens 0.7 mm thick and 10 mm in diameter. Both sides of the Initial LiSi Block specimens’ surface were polished using #1000 SiC paper. Dentine base specimens were prepared by injecting material in transparent 3D-printed moulds, 2 mm thick and 10 mm in diameter, kept on a glass slab and covered with a celluloid strip. Composite was light-cured for 10 s using the same light-curing unit (SmartLite) at a 1 mm distance. Each specimen was removed from its mould and covered with a double layer of nail varnish by applying the varnish to the bottom surface and the sides and leaving the top surface for cementation. The purpose of the varnish application was to avoid penetration of the staining medium into the dentine base.

The veneer–cement–base assembly specimens were prepared in 2.9 mm thick 3D-printed moulds (2 mm dentine base, 0.2 mm resin cement and 0.7 mm veneer) with side perforations to allow the evacuation of excess resin cement. A G-Multi Primer was applied on the veneer side according to manufacturer’s recommendations. Cement was injected onto the dentine base, pressed with the LiSi veneer using finger pressure until seated in the mould. As much as possible excess was removed before light-curing using a brush. Light-curing was performed for 10 s at a 1 mm distance from the surface of the veneer. After curing, sof-lex discs (light red, orange and yellow) for 10 s each were used to remove any remaining cement excess. Specimens were stored in distilled water at 37 °C for 24 h prior to baseline colour measurement.

### 2.2. Staining

Specimens prepared of resin cements only were immersed entirely in the staining medium. One bag of Lipton’s black tea that was immersed in 200 mL of boiling water was kept for 5 min. Each specimen was immersed in 10 mL of the staining medium.

**Figure 1:**
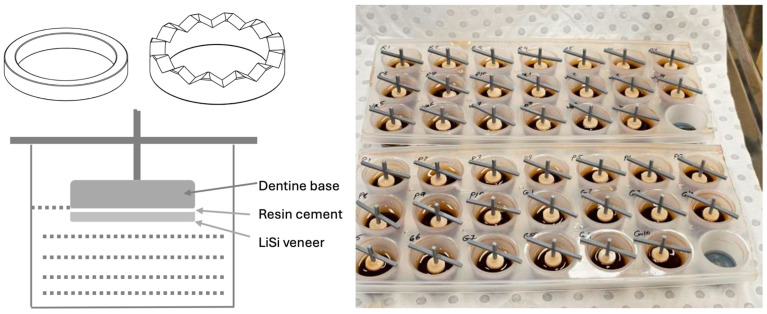
Three-dimensional-printed moulds for standardized specimen preparation and cemented veneer specimens immersed in black tea.

Specially designed racks were used for cement-only specimens to allow maximum exposure to the staining medium. A 3D-printed handle was used to fixate cemented veneer specimens to the bottom side of the varnish-coated dentine base using a small amount of flowable composite without adhesive for easy removal. The specimens were placed facing upside down in a staining container with the handle keeping the specimen suspended so that only part of the specimen could be exposed to the staining solution (Figure 1). The amount of tea was carefully measured to cover only the veneer and cement portion of the specimen, leaving the dentine base outside the tea. This was verified visually under magnification. Containers were closed to prevent evaporation of the tea. Specimens were stored in an incubator at 37 °C for 72 h, with daily changes in the tea. Changes in pH were not monitored over time as a previous study showed virtually no changes after 4 h or 24 h [23]. Staining of 72 h corresponds to a year of service with about 12 min of daily exposure. After staining, the handle was easily removed using shear force.

### 2.3. Colour and Translucency Parameter

Colour measurements were performed initially and after staining using a spectrophotometer (CM-25 Konica Minolta, Tokyo, Japan) with the accompanying software for CIELab values. Calibration was performed against a standard white/black background. Resin cement specimens were placed with their bottom parts (further from the light tip) facing the background. Cemented LiSi veneers were placed with their dentine base facing the background. CIEDE2000 colour changes (*∆E_00_*), the translucency parameter (*TP_00_*) and TP changes (*∆TP_00_*) were calculated using the following formulae:

$${\Delta E}_{00}={\left[{\left(\frac{∆L^{\prime }}{K_{L\;}S_{L}}\right)}^{2}+{\left(\frac{∆C^{\prime }}{K_{C\;}S_{C}}\right)}^{2}+{\left(\frac{∆H^{\prime }}{K_{H\;}S_{H}}\right)}^{2}+R_{T}\left(\frac{∆C^{\prime }}{K_{C}S_{C}}\right)\left(\frac{∆H^{\prime }}{K_{H}S_{H}}\right)\right]}^{\frac{1}{2}}$$

$${TP}_{00}=\;{\left[{\left(\frac{{L^{\prime }}_{B\;}-{L^{\prime }}_{W}}{K_{L}S_{L}}\right)}^{2\;}+{\left(\frac{{C^{\prime }}_{B}-{C^{\prime }}_{W}}{K_{C}S_{C}}\right)}^{2\;}+{\left(\frac{{H^{\prime }}_{B}-{H^{\prime }}_{W}}{K_{H}S_{H}}\right)}^{2\;}\;+RT\;\left(\frac{{C^{\prime }}_{B}-{C^{\prime }}_{W}}{K_{C}S_{C}}\right)\left(\frac{{H^{\prime }}_{B}-{H^{\prime }}_{W}}{K_{H}S_{H}}\right)\right]}^{\frac{1}{2}}$$

$$\Delta {TP}_{00}={TP}_{00}\left(stained\right)-{TP}_{00}\left(initial\right)$$

where *L*, *C* and *H* denote lightness, chroma and hue, respectively, against white (*W) and black (*B) backgrounds, and ∆*L*′, ∆*C*′, ∆*h*′ are differences between the corresponding colour coordinates, computed based on the uniform colour space used in CIEDE2000. RT is the rotation function accounting for the interaction between hue and chroma differences in the blue region. *S_L_*, *S_C_*, *S_H_* adjust the total colour difference for variation in the location of the colour difference sample over the *B* and *W* backgrounds in *L*′, *a*′, *b*′ coordinates and the parametric factors, *K_L_*, *K_C_*, *K_H_*, are correction terms for experimental conditions.

### 2.4. Sorption and Solubility

The sorption and solubility of resin cements (N = 5/group) were tested using the 10 × 2 mm resin cement specimens, prepared as explained in Section 2.1. Sorption and solubility testing was performed according to the 4049:2019 standard [24], however, modified with respect to the immersion solution and duration of exposure so as to mimic the experimental conditions for colour and translucency measurements. Sorption (*Wsp*) and solubility (*Wsl*) were calculated using the following equations, based on specimen mass differences and volume:

$$Wsp=\frac{m2-m3}{V}$$

$$Wsl=\frac{m1-m3}{V}$$

The specimens were kept in a desiccator incubator and weighed on an analytical balance (d = 0.1 mg, Sartorius) until constant mass, *m*1, was achieved; i.e., the mass loss of each specimen was not more than 0.1 mg different in any 24 h period. The dimensions of each specimen (diameter and thickness) were measured using a digital calliper to an accuracy of 0.1 mm and the volume (*V*) of each specimen was then calculated in mm^2^.

The specimens were then placed on a rack so that the entirety of each specimen can be exposed to the solution, immersed in Lipton’s black tea (10 mL per specimen) and kept at 37 °C for 72 h. After 72 h, the specimens were rinsed with water, blot-dried and weighed to record the mass *m*2. After the weighing, the specimens were reconditioned to constant mass in the desiccator incubator and periodically weighed until achieving the constant mass *m*3.

### 2.5. Statistical Analysis

Data were analyzed using one-way ANOVA with Tukey’s post hoc test, correlation and regression analyses (GraphPad Prism Version 10.1.0(264)). Normal distribution was checked using the Kolmogorov–Smirnov test, and equal variances were tested using the Levene’s test. All tests were performed with a level of significance set at alpha = 0.05.

## 3. Results

**Figure 2:**
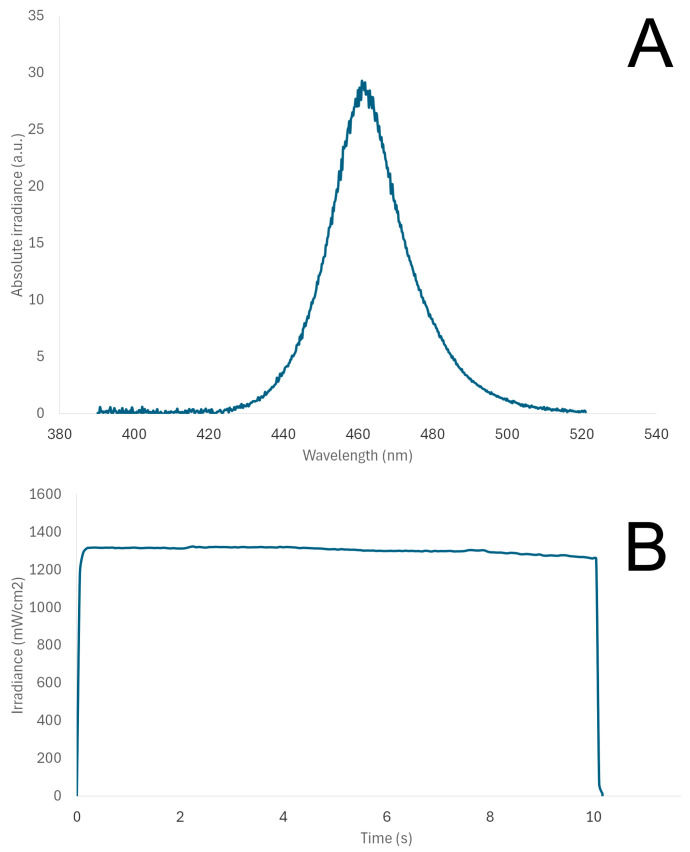
Wavelength (**A**) and irradiance (**B**) of the SmartLite Pro light-curing unit during light exposure.

Figure 2 presents the spectral analysis of the SmartLite Pro light-curing unit, used for light-curing specimens in this study. Maximum irradiance was detected at 461 nm, whilst the mean irradiance was 1303.8 ± 18.5 mW/cm^2^, and the total energy delivered was 13.1 J/cm^2^.

### 3.1. Resin Cements

**Figure 3:**
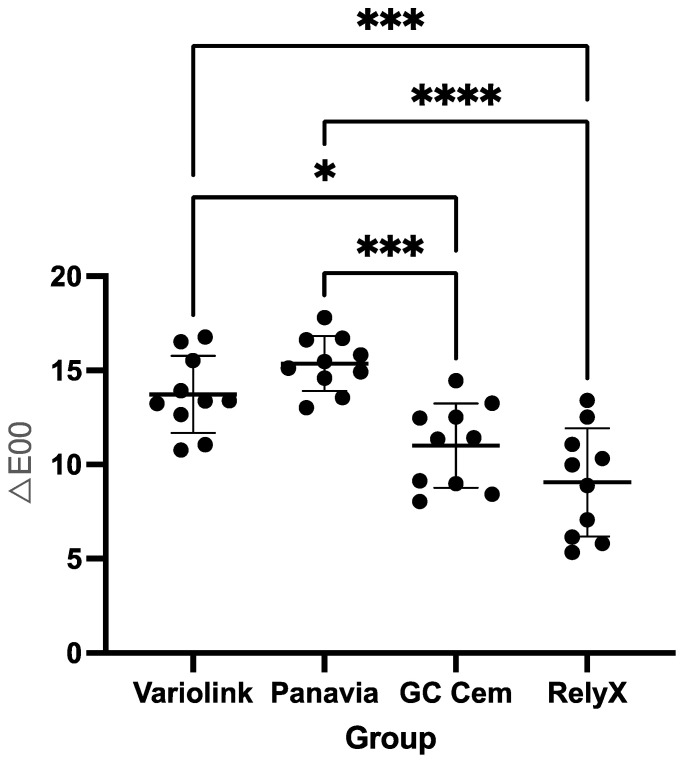
Colour differences (*∆E_00_*) of the tested resin cements after staining in black tea (mean and standard deviation). Groups connected with lines and indicated by asterisks are significantly different (*p* < 0.05). Black dots indicate individual values; horizontal lines indicate mean and SD values.

Regarding colour changes (*∆E_00_*), GC Cem (mean ± SD: 11.0 ± 2.2) showed no statistically significant difference (*p* = 0.2166) from RelyX (9.1 ± 2.9). Both GC Cem and RelyX showed significantly lower (*p* < 0.001) *∆E_00_* after staining in black tea compared to Variolink (14.0 ± 2.1) and Panavia (15.0 ± 1.5) (Figure 3).

**Figure 4:**
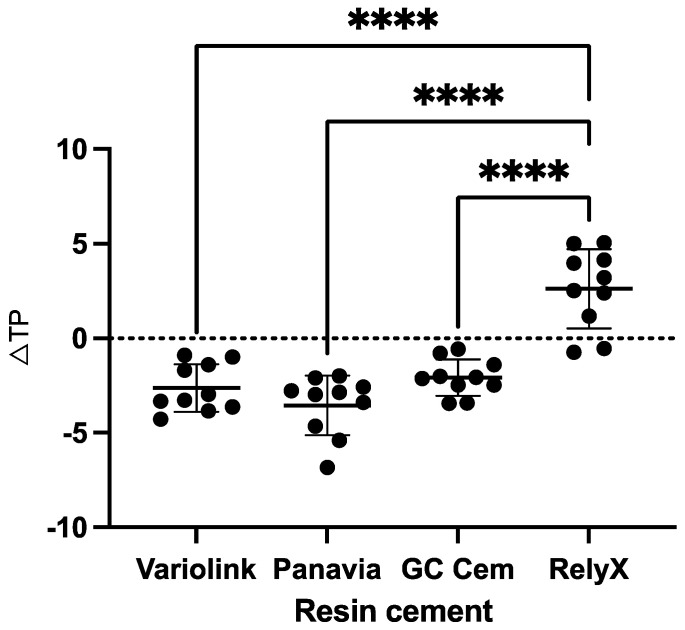
Changes in *TP_00_* of the tested resin cements after staining in black tea (mean and standard deviation). Groups connected with lines and indicated by asterisks are significantly different (*p* < 0.05). Black dots indicate individual values; horizontal lines indicate mean and SD values. Negative values indicate decrease in *TP_00_* after staining.

Regarding initial translucency (*TP_00_*), the highest values were measured for Panavia (mean ± SD: 13.37 ± 0.73), followed by Variolink (10.61 ± 0.70), GC Cem (7.83 ± 0.71) and RelyX (7.20 ± 0.90). Regarding translucency changes (*∆TP_00_*) after staining, GC Cem (−2.10 ± 0.97), Variolink (−2.6 ± 1.3) and Panavia (−3.6 ± 1.6) showed a decrease in *TP_00_* (becoming more opaque) and were all significantly different from RelyX (2.6 ± 2.1), which showed an increase in *TP_00_* (becoming more translucent) after staining (*p* < 0.001). There was no difference in *∆TP_00_* between GC Cem, Variolink and Panavia (*p* > 0.05) (Figure 4).

**Figure 5:**
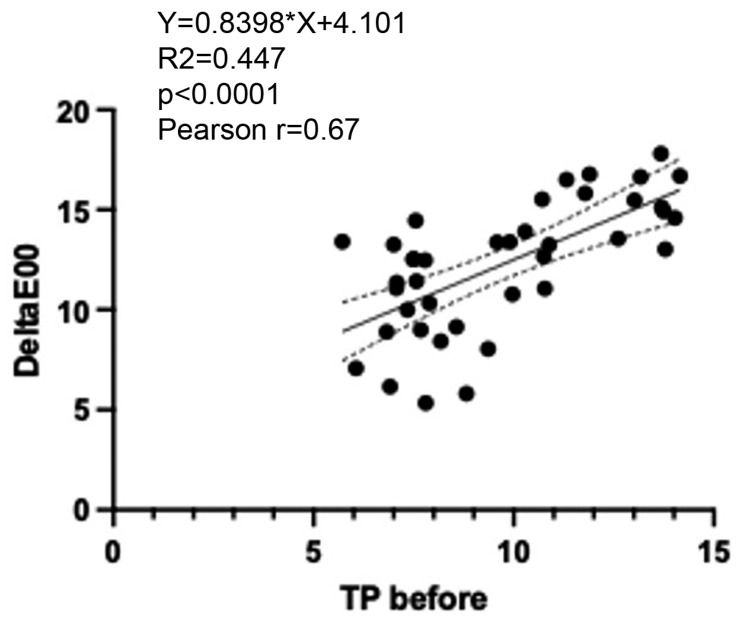
Pearson correlation (r) and linear regression analysis of initial translucency (*TP_00_*) and colour changes (*∆E_00_*) of resin cements.

There was a significant positive correlation between initial translucency and subsequent colour changes in resin cements with the Pearson correlation coefficient as r = 0.67 (*p* < 0.0001). Furthermore, linear regression analysis showed that colour changes in the tested resin cements could be predicted to a certain extent based on the initial translucency of the cement Y = 0.8398 × X + 4.101 (R^2^ = 0.4477; *p* < 0.0001) (Figure 5).

**Figure 6:**
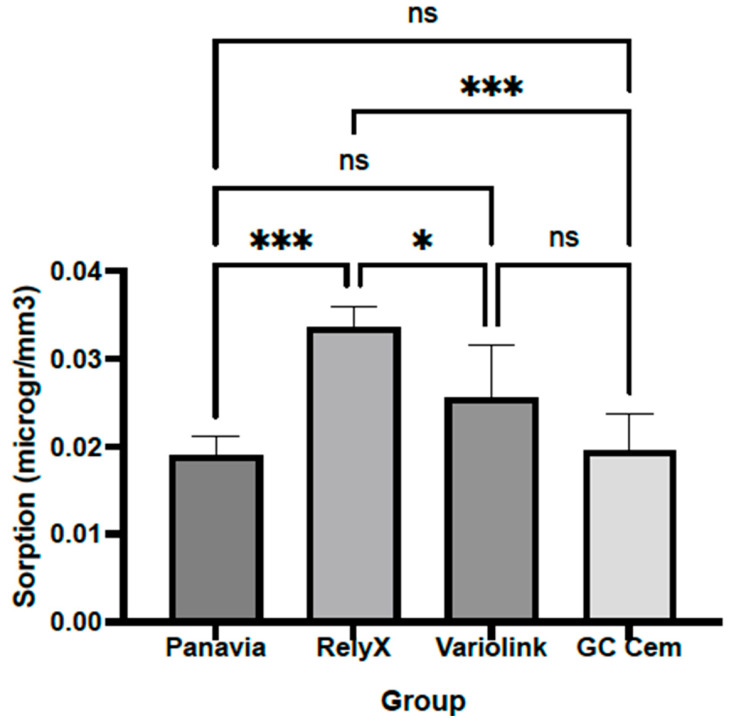
Sorption of resin cements after 72 h exposure to black tea. Columns indicate means, and bars indicate SD values. Groups connected with lines and indicated by asterisks are significantly different (*p* < 0.05). Abbreviation: ns—not significant.

The sorption of RelyX (0.0336 ± 0.0024 μg/mm^3^) was significantly higher than that of other tested resin cements, RelyX vs. Panavia (0.0190 ± 0.0021 μg/mm^3^; *p* = 0.0002), RelyX vs. GC Cem (0.0195 ± 0.0042 μg/mm^3^; *p* = 0.0004) and RelyX vs. Variolink (0.0256 ± 0.0058 μg/mm^3^; *p* = 0.0261). There was no statistically significant difference in sorption among other cements (*p* > 0.05) (Figure 6). Post hoc power analysis based on the sample size based on the ISO standard and obtained mean and SD data for sorption showed that the power was 0.916.

**Figure 7:**
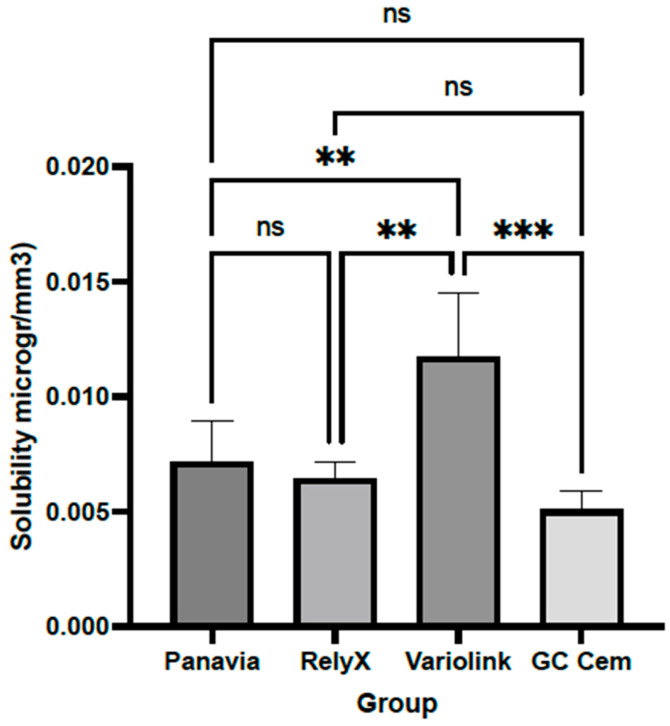
Solubility of resin cements after 72 h exposure to black tea. Columns indicate means, and bars indicate SD values. Groups connected with lines and indicated by asterisks are significantly different (*p* < 0.05). Abbreviation: ns—not significant.

The solubility of Variolink (0.0117 ± 0.0028 μg/mm^3^) was significantly higher than that of other tested resin cements, Variolink vs. Panavia (0.0072 ± 0.0018 μg/mm^3^; *p* = 0.0047), Variolink vs. GC Cem (0.0051 ± 0.0008 μg/mm^3^; *p* = 0.0002) and Variolink vs. RelyX (0.0064 ± 0.0007 μg/mm^3^; *p* = 0.0014). There was no statistically significant difference in solubility among other cements (*p* > 0.05) (Figure 7). Post hoc power analysis based on the sample size based on the ISO standard and obtained mean and SD data for solubility showed that the power was 0.843.

There was no correlation between the sorption and solubility of resin cements (r = 0.2372, *p* = 0.3282). Correlation analysis showed no significant correlation between the sorption and *∆E_00_* of cements (r = −0.2472, *p* = 0.2933). Furthermore, there was no correlation between solubility and the *∆E_00_* of cements (r = 0.4245, *p* = 0.0621).

### 3.2. Cemented LiSi Veneers

**Figure 8:**
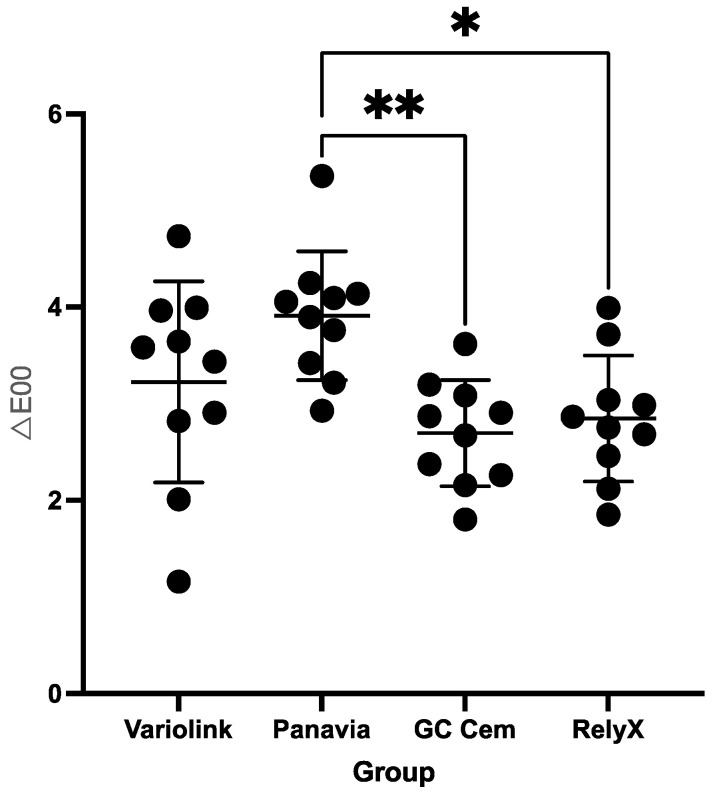
Colour differences (*∆E_00_*) of the veneer–cement–base specimens after staining in black tea (mean and standard deviation). Groups connected with lines and indicated by asterisks are significantly different (*p* < 0.05). Black dots indicate individual values; horizontal lines indicate mean and SD values.

Regarding colour changes (*∆E_00_*), there was no statistically significant difference between GC Cem (2.70 ± 0.5) and RelyX (2.8 ± 0.6) (*p* = 0.9687). However, both GC Cem and RelyX groups had a significantly lower *∆E_00_* compared to the Panavia group (3.9 ± 0.7) (GC Cem vs. Panavia *p* = 0.0048 and RelyX vs. Panavia *p* = 0.0158). The difference in *∆E_00_* between the Variolink group (3.2 ± 1.0) and GC Cem or RelyX groups was not significant (*p* > 0.05) (Figure 8).

**Figure 9:**
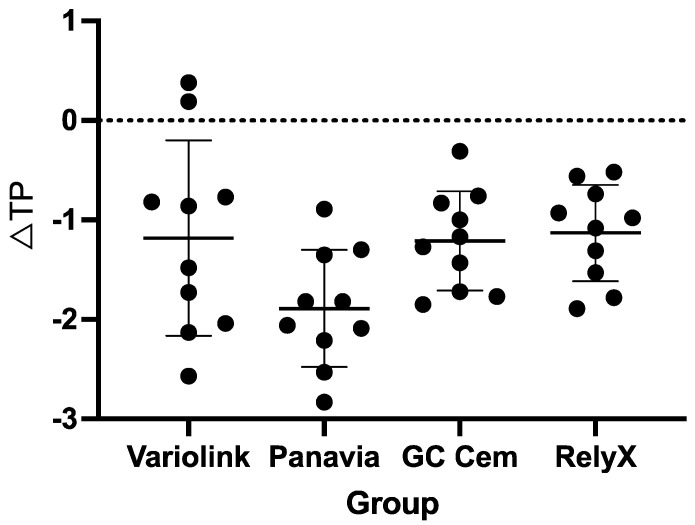
Changes in translucency parameter (∆*TP_00_*) of the cemented LiSi veneer specimens after staining in black tea (mean and standard deviation). Black dots indicate individual values; horizontal lines indicate mean and SD values. Negative values indicate a decrease in *TP_00_* after staining.

Regarding translucency changes (*∆TP_00_*), initial translucency decreased in all cemented LiSi veneer groups after staining. No significant differences were observed between the tested groups of veneer–cement–base specimens (*p* > 0.05). The values were in the range of −1.1 ± 0.5 for RelyX to −1.9 ± 0.6 for Panavia (Figure 9).

Correlation analysis showed no significant correlation between sorption and the *∆E_00_* of cemented LiSi veneers (r = −0.0950, *p* = 0.6905). There was no correlation between solubility and the *∆E_00_* of cemented LiSi veneers (r = 0.2516, *p* = 0.2846).

**Figure 10:**
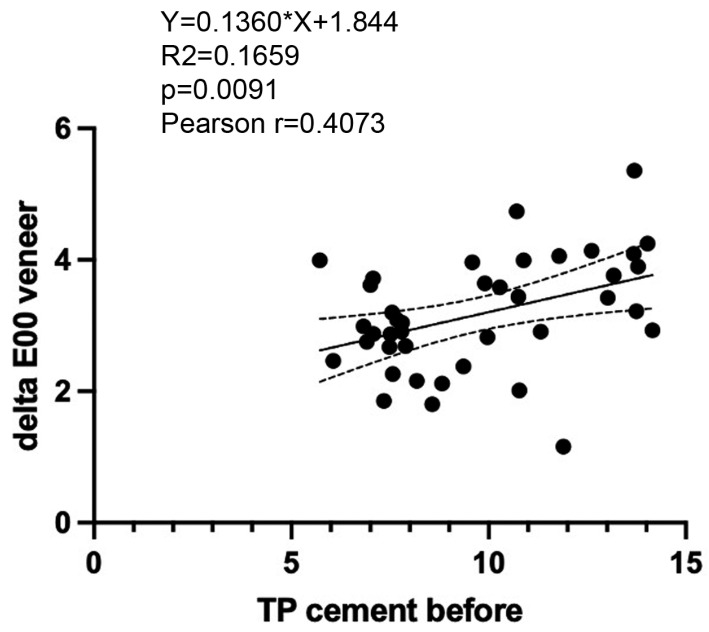
Pearson correlation (r) and linear regression analysis of initial translucency (*TP_00_*) of resin cements and colour changes (*∆E_00_*) of cemented LiSi veneers.

There was a significant positive correlation between the initial translucency of resin cements and subsequent colour changes in cemented LiSi veneers with the Pearson correlation coefficient as r = 0.4073 (*p* = 0.0091). Furthermore, linear regression analysis showed that colour changes in cemented LiSi veneers could be predicted to a certain extent based on the initial translucency of the cement Y = 0.1360 × X + 1.844 (R^2^ = 0.1659; *p* = 0.0091) (Figure 10).

**Figure 11:**
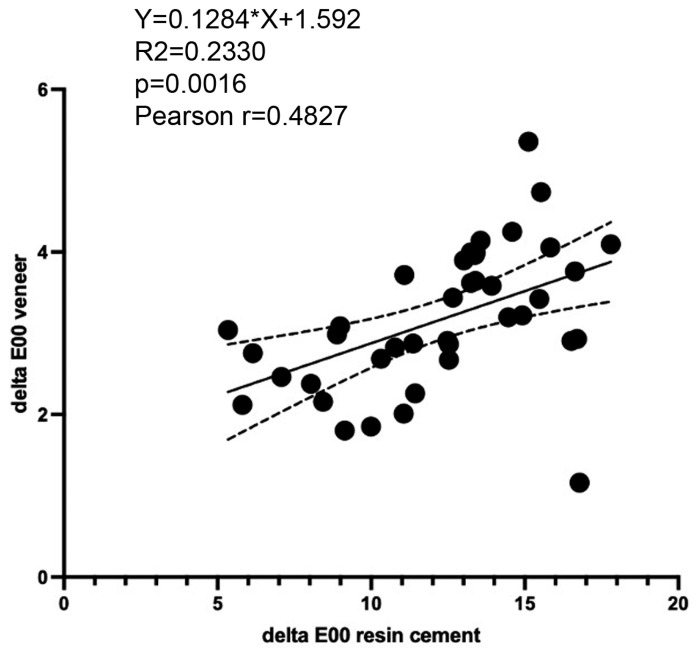
Pearson correlation (r) and linear regression analysis of colour changes (*∆E_00_*) of resin cements and colour changes (*∆E_00_*) of cemented LiSi veneers.

There was a significant positive correlation between the colour changes *∆E_00_* of resin cements and colour changes *∆E_00_* of cemented LiSi veneers with the Pearson correlation coefficient as r = 0.4827 (*p* = 0.0016). Furthermore, linear regression analysis showed that colour changes in cemented LiSi veneers could be predicted to a certain extent based on the colour changes in the cement Y = 0.1284*X + 1.592 (R^2^ = 0.2330; *p* = 0.0016) (Figure 11).

## 4. Discussion

The first hypothesis (H_0_#1) was rejected as significant differences were found in colour and translucency, sorption and the solubility of resin cements after staining. The second hypothesis (H_0_#2) was only rejected for colour and not for translucency as the former significantly differed in cemented LiSi veneers after staining, whilst the latter showed no statistically significant differences. The third null hypothesis (H_0_#3) was also rejected as a significant positive correlation was observed between the initial translucency of resin cements and subsequent colour changes in cemented LiSi veneers as well as between the colour changes *∆E_00_* of resin cements and colour changes *∆E_00_* of cemented LiSi veneers. The fourth null hypothesis (H_0_#4) was upheld as no significant relationship was found between the sorption/solubility of resin cements and colour changes *∆E_00_* of resin cements/cemented LiSi veneers.

The baseline measurements of *L*, *a* and *b* values only position a specimen in the colour coordinate system. This initial position does not give sufficient information on materials’ performance unless it is compared to another element. In dentistry, the matching of colour/translucency (and other optical properties) between a specimen and tooth is important for initial aesthetics. Changes in these properties between an initial value and a value after staining or ageing are important for long-term aesthetic outcomes. In both instances, perceptibility and acceptability thresholds are used to contextualize observed changes irrespective of the initial position of a material in the colour coordinate system. As the present study only focused on materials, the tested hypotheses focused on colour and translucency changes after staining to indicate staining resistance of the tested materials under the same experimental conditions.

When resin cements were tested alone, a common approach was adopted that entailed the full immersion of cement specimens in a staining medium. This was performed in previous studies [11,15,16,17,18,19,20,21] and allows data comparison to some extent. Resin cements are prone to discolouration, with a great range of colour differences reported after storage in water, from *∆E_ab_* 0.5–2.3 [16,18] to *∆E_ab_* 4.3–16.9 [19]. Differences are related to materials and experimental conditions, varying in the length and temperature of exposure. Staining media are, as would be expected, more potent discolourants of resin cements than water [16,18]. Red wine has shown significantly greater staining of resin cements than coffee, cola or tea, with coffee, cola and tea showing material-dependent effects [18,20,21]. Similarly, red wine and coffee were shown to induce greater differences than other staining media in optical properties in resin composite restorative materials [25,26,27]. Black tea, or tea in general, is not commonly used for staining resin cements [17,20,21]. Further, a range of exposures can be found in the literature, ranging from 1 day to several months [11,15,16,17,18,19,20,21], which is consistent with a variety of exposure scenarios for resin composite materials in general [28]. Due to a lack of standard and wide dietary habits, it is entirely arbitrary for researchers to select an exposure scenario.

Testing commercially available materials does not allow much novelty due to the presence of a number of studies in the field. However, each study brings new information in relation to the specific testing conditions. This is of particular importance in staining studies due to the huge variety of individual dietary habits and limited laboratory testing ability to cover this range. The present study adds further information on the relationships between translucency and colour changes, allowing better understanding of the staining mechanisms, the possibility to predict changes in relation to initial properties and the clinical optimization of material selection.

In the present study, staining in black tea had a considerably more profound effect on colour than on the translucency of resin cements. Although GC Cem and RelyX exhibited lower *∆E_00_* compared to Variolink and Panavia, the *∆E_00_* of all tested resin cements were much higher than the perceptibility and acceptability thresholds for tooth-coloured materials [29]. This can be attributed to relatively high resin content required to maintain the low viscosity of resin cements. Conversely, the *∆E_00_* of cemented LiSi veneers were much lower than the *∆E_00_* of resin cements alone and in the range of around 2.5 and 3.9, which mostly corresponds to the mismatch type [a] and, to a lesser extent, type [b] [29]. Translucency differences *∆TP_00_* of the tested resin cements were in the range of 2.1–3.6, which corresponds mostly to mismatch type [a], whilst the *∆TP_00_* of the cemented veneers was in the acceptable match range (1.1 and 1.9) [29].

Less colour change was detected in the LiSi veneers luted with GC Cem and RelyX compared to Panavia, which was in accord with the findings for resin cements alone. Furthermore, a significant positive correlation was found between the *∆E_00_* of resin cements and *∆E_00_* of cemented LiSi veneers. This finding suggests that the testing staining capacity of resin cements by a full immersion of cement specimens in staining media could be a simple screening method prior to a more complex experimental design for testing cemented ceramic materials.

Cemented LiSi veneers in the present study were exposed to the staining medium only in the part corresponding to the veneer and cement whilst the dentine base was kept out of the medium and protected by nail varnish. This approach was taken to mimic the clinical conditions and to exclude the effect of staining the dentine base, which would be inevitable in the case of full specimen immersion. In previous studies, in which the optical properties of cemented veneers were tested, no staining/ageing was attempted [6,7,8], or the specimens were fully immersed in water during extended storage [10].

Generally, the same mechanisms of staining are responsible for the staining of resin cements as for resin composite restorative materials. Intrinsic factors involve the sorption of the staining medium, leading to the swelling and plasticization of resin matrices, further dependent on the filler content and initiator system [6,30,31]. Aromatic amine accelerators and inhibitors in resin composite cements may cause colour changes due to oxidation reactions, leading to discolouration from yellow to brown over time. Aliphatic amines in light curing systems were shown to be more colour-stable than aromatic amines in chemical curing systems, and newer systems without benzoyl peroxide/amine redox initiators showed less colour change [13,14,32]. Extrinsic factors are related to characteristics of the staining medium, namely, pigment penetration into the polymer network [33] and adsorption on the material surface, which may be mitigated by re-polishing [25].

GC Cem and RelyX in the present study showed better staining resistance than Variolink. A recent study also showed significantly less colour change in another cement from the RelyX “family” (self-adhesive RelyX U200) compared to Variolink after a day and a week of staining in black tea [17].

The translucency of resin cements tends to decrease after ageing/staining, as was observed in the present study and in previous studies using different resin cements [18,34,35]. One exception appeared to be RelyX, which showed increased translucency in the present study. This finding can be related to increased sorption observed for RelyX, significantly higher than that of other tested resin cements, even after 72 h of storage in the staining medium. RelyX contains components that contributed to increased hydrophilicity, such as HEMA, TEGDMA, as well as a newly introduced amphiphilic monomer and a novel amphiphilic redox initiator system. The latter two components are intended to improve cement interaction with hydrophilic dentine, the formation of a highly cross-linked polymer network and improved bond strength [36]. Changes in translucency may affect the masking ability of resin cement in relation to the substrate, resulting in inferior aesthetics over time. This is of particular importance in thin and translucent veneers as it was shown that the masking of substrates was associated with low translucency veneers and opaque resin cement [37].

Whilst the highest sorption was found in RelyX as discussed above, Variolink showed significantly greater solubility than other tested resin cements. Large variations for the solubility of Variolink Esthetic can be found in the literature following the same ISO 4049 standard [38,39,40]. These findings could be related to differences in the quality of polymerization in different studies as different light-curing units and curing protocols were used. It should be noted that both the sorption and solubility of resin cements were well within the ISO 4049 specifications, sorption lower than 40 μg/mm^3^ and solubility lower than 7.5 μg/mm^3^ after water storage for seven days [38]. Despite shorter exposure of 3 days in black tea instead of a week in distilled water, the sorption of resin cements in the present study was well below 1 μg/mm^3^, and the solubility was below 0.02 μg/mm^3^. In clinical settings, a much smaller portion of cement is directly exposed to the oral environment, indicating that sorption and solubility are even less affected than in the extreme experimental conditions of in vitro testing. As it is not possible to test the effects of sorption and solubility clinically, it would be prudent to modify the standard to include long-term effects of these properties on the restoration durability.

The present study showed no significant correlation between the sorption and solubility of tested resin cements and colour differences in the same staining medium (black tea). This highlights the fact that colour differences in resin cements are likely material- and medium-dependent. It should be noted that different types of interactions occur with water and coloured beverage staining. While sorption and solubility can cause internal changes, namely, swelling and index refraction variations affecting colour [16], staining in coloured media is likely more dependent on surface interactions and pigment penetration, which may explain the lack of correlation between sorption/solubility and colour differences in resin cements and cemented veneers.

Significant positive correlation was found between the initial translucency of resin cements and their colour differences after staining as well as those of cemented LiSi veneers, albeit to a smaller extent as indicated by R^2^ values. Cements with higher initial translucency are more prone to staining than those with lower initial translucency. This is of importance in veneer restorations, which themselves are very thin and highly translucent, emphasizing the effect of underlying cement on the long-term aesthetic appearance of the restoration. In the present study, Panavia showed the highest initial *TP_00_* whilst GC Cem and RelyX showed the lowest initial *TP_00_*. A low initial *TP_00_* of GC Cem and RelyX was associated with less colour change in these resin cements alone and GC Cem- and RelyX-cemented veneers. Clinicians should avoid highly translucent cements and opt for cements with lower initial translucency for patients with a high intake of coloured food and beverages to minimize discolouration, maintaining the aesthetic effects of veneer restorations.

Limitations of the present study include the use of one dentine base shade, which narrows the result range but also helps standardize the approach and allow the comparison of resin cements. To control the number of confounding factors, one thickness of both the veneer and resin cement was tested. Cement thickness was based on common clinical scenarios [7,41], and veneer thickness was within the manufacturer’s recommended thickness range [42]. Another limitation is that no adhesive/primer was used with resin cements when applied to the dentine base. Each resin cement is recommended with a different primer/adhesive for the tooth surface. Given that no teeth were used for dentine base and to avoid multiple confounding factors, an etching and adhesive application to the composite dentine base was not performed. This standardized approach allowed us to focus only on the effects of resin cements, on the colour and translucency of the cemented veneers.

Further research should include different specimen thickness (both cement and veneer), curing methods, multiple staining media and exposure scenarios to ascertain the robustness of these materials and correlations of tested properties in order to optimize clinical practises. An expanded methodological approach will require new sample size calculations to enhance the statistical power and reliability of the results.

## 5. Conclusions

Based on the present findings, it can be concluded that resin cements affect the colour but not translucency of cemented LiSi veneer restorations after staining in black tea. Colour changes in cements and cemented veneer restorations were associated with an initial translucency of the tested cements but not with their sorption and solubility. Colour changes in resin cements positively correlated with colour changes in cemented veneer restorations. G-CEM ONE and RelyX Universal showed less colour change than Panavia V5 and Variolink Esthetic. G-CEM ONE- and RelyX-cemented veneers showed less colour change than Panavia-cemented veneers.

## Figures and Tables

**Table 1:** Materials used in this study.

Material (Code)	Manufacturer	Shade	Adhesion Mode	Composition
			Curing Mode	
Variolink Esthetic (Variolink)	Ivoclar Vivadent, Schaan, Liechtenstein	Neutral	Adhesive	Urethane dimethacrylate (UDMA), glycerin-1.3-dimethacrylate, 1,10-decandiol dimethacrylate, ytterbium trifluoride and mixed spheroidal oxide fillers (particle size 0.04 to 0.2 µm, with an average size of 0.1 µm), butylated hydroxytoluene (BHT), initiators, stabilizers, pigments
			Dual-cured	
Panavia V5 (Panavia)	Kuraray Noritake, Tokyo, Japan	Clear	Adhesive	Paste A/Paste B Bisphenol A diglycidylmethacrylate (Bis-GMA), triethyleneglycol dimethacrylate (TEGDMA), hydrophobic aromatic dimethacrylate, hydrophilic aliphatic dimethacrylate, initiators, accelerators, silanated barium glass filler (particle size range 0.01–12 µm), silanated fluoroalminosilicate glass filler, colloidal silica bisphenol A Bis-GMA, hydrophobic aromatic dimethacrylate, hydrophilic aliphatic dimethacrylate, silanated barium glass filler, silanated alminium oxide filler, accelerators, dl-Camphorquinone, pigments
			Dual-cured	
G-CEM ONE (GC Cem)	GC Corporation, Tokyo, Japan	Translucent	Self-adhesive	Paste A/Paste B 2-Hydroxy-1,3 dimethacryloxypropane, urethane Dimethacrylate (UDMA), titanium dioxide, silicon dioxide and fluoro-almino-silicate glass, 6-tert-butyl-2,4-xylenol, diphenyl(2,4,6-trimethylbenzoyl)phosphine oxide Urethane dimethacrylate (UDMA), 2-Hydroxy-1,3 dimethacryloxypropane, methacryloyloxydecyl dihydrogen phosphate, α,α -dimethylbenzyl hydroperoxide, 6-tert-butyl-2,4-xylenol, and silicon dioxide
			Dual-cured	
RelyX Universal (RelyX)	3M, Seefeld, Germany	Translucent	Universal	Base: phosphorus oxide, silane, terimethoxyctyl-,hydrolysis product with silica, t-Amyl hydroxiperoxide, n2,6-di-tert-butyl-p-cresol, 2-HEMA, methyl methacylate, acetic acid, copper salt, monohydrate Catalyst: diurethanedimethacrylate, ytterbium fluoride, glass powder, surface modified with 2-propenoic acid, 2 methyl-3-(trimethoxysilyl)propyl ester and phenyltrimethoxy silane, TEGDMA, L-ascorbic acid, 6-hexadecanoate, hydrate, silane, trimethoxyoctyl, hydrolysis product with silica, 2-HEMA, titanium dioxide, triphenyl phosphite
			Dual-cured	
Initial LiSi Block (LiSi)	GC Corporation, Tokyo, Japan	A1 HT	CAD/CAM block	Fully crystallized lithium disilicate glass ceramic
G-aenial Universal Injectable (G-aenial)	GC Corporation, Tokyo, Japan	AO3	Injectable resin composite	(Octahydro-4,7-methano-1H-indenediyl)bis(methylene) bismethacrylate, 2,2-dimethyl-1,3-propanediyl bismethacrylate, 1,3,5-Triazine-2,4,6-triamine, polymer with formaldehyde, diphenyl(2,4,6-trimethylbenzoyl)phosphine oxide, 2,2′-ethylenedioxydiethyl dimethacrylate, 2-(2H-benzotriazol-2-yl)-p-cresol, Urethane dimethacrylate (UDMA), 6-tert-butyl-2,4-xylenol, ultrafine barium glass fillers (150 nm, ~69 wt%)

## Data Availability

The data presented in this study are available upon request from the corresponding author due to the need to consult the funder.
